# Supplementary material for: The Impact of Indoles Activating the Aryl Hydrocarbon Receptor on Androgen Receptor Activity in the 22Rv1 Prostate Cancer Cell Line
Source: Int J Mol Sci. 2022 Dec 28;24(1):502. doi: 10.3390/ijms24010502 (PMC9820252; doi:10.3390/ijms24010502)
Supplement: Supplementary file 1 [file ijms-24-00502-s001.zip › ijms-2049810-supplementary.pdf]

Supplement S1. Stable transfection of 22Rv1 cell line

Initially, a cellular system was established to monitor AhR transcriptional activity in the 22Rv1 cell line. Therefore, a stably transfected 22AhRv1 cell line with the luciferase reporter gene was constructed.

22Rv1 cells were transfected with the plasmid pGL4.27 [luc2P/minP/Hygro] designed by Novotna *et al.* 2011 [1]. Cells were seeded at the density 5 x 10^4 cells in 1 well of 12-well plate in 1 ml of the RPMI medium and transfected with pGL4.27-DRE reporter plasmid (200 ng per well) using FuGENE® Transfection Kit (Promega), according to protocol. Cells were maintained in transfection medium in a humidified incubator for 48 h. Subsequently, the culture medium was replaced with selection medium containing Hygromycin B (Hyg B) inhibitory concentration 25 for 4 days (IC_{25} = 60 μg/ml). The selection pressure was then increased by adding selection medium with 50 % inhibitory concentration (IC_{50} = 110 μg/ml) of HygB. The IC_{50} selection medium was renewed every 3-4 days for 4 weeks, when a polyclonal population was selected.

Subsequently, cells of the polyclonal population were seeded in 10 mm culture plates at the density of 1000 cells per plate. Cells were cultured for another 3 weeks in selection medium (IC_{50}), until colonies of the monoclonal population were visible. Monoclonal colonies were collected, labeled and tested with the Reporter gene assay (RGA) for response to AhR ligand TCDD. Ten different monoclonal populations were isolated for the 22Rv1 transfected cell line. For further characterization, clone 10 of 22Rv1 transfected cell line (titled as **22AhRv1**) was selected.

The novel prostate-specific stably transfected cell line 22AhRv1 was subjected to further characterization, such as dose-dependent response for endogenous and exogenous AhR ligands (TCDD, FICZ, B[a]P), time-dependent analysis of luciferase inducibility, and freeze-thaw cycle monitoring. The results were evaluated with Infinite M200.

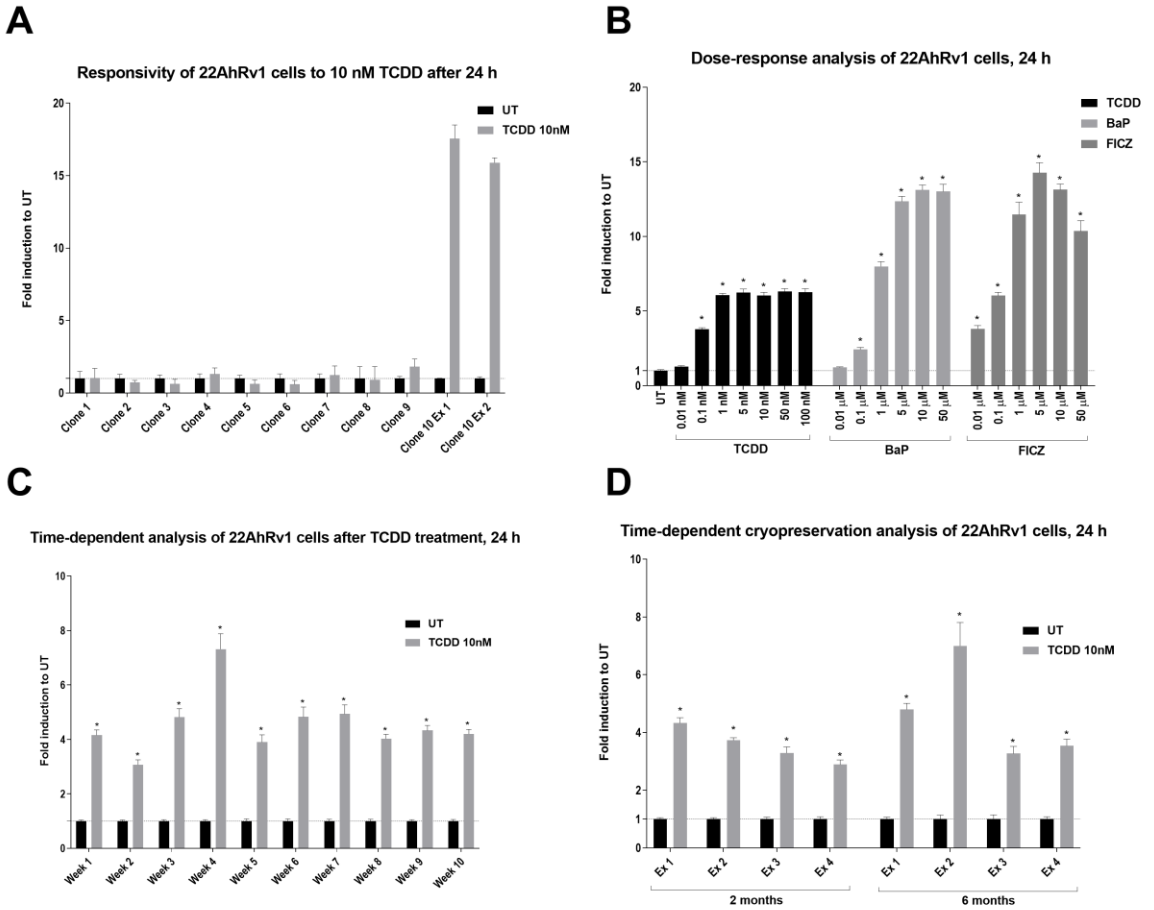

Supplement S1: **Characterization of the newly developed prostate-specific AhR reporter cell line**. 22Rv1 cells were transfected with the reporter plasmid pGL4.27 [luc2P/minP/Hygro]. Monoclonal population was isolated, labelled and tested for response to TCDD (10 nM) by RGA (A). For further characterization, Clone 10 was selected and titled as **22AhRv1** and tested for dose-dependent response of endogenous and exogenous ligands (TCDD, FICZ, B[a]P) of AhR (B). 22AhRv1 cell line was further evaluated for long-term stability of luciferase inducibility (C) and freeze-thaw cycles (D). The results are expressed as fold induction to untreated (UT; DMSO-treated cells).

Supplement S2. Cytotoxycity and proliferation assays

22Rv1 cells were seeded in 96-well plates at a density of 2.5 x 10^4 cells per well in volume 200 µl and stabilized until the next day. Subsequently, cells were treated with increasing concentrations (final concentration 1-100 µM) of the tested indoles (***Table 2***) and controls – DMSO (untreated/UT; 0.1 %; v/v) or doxorubicin (positive control; final concentration 20 µM) for 24 h.

a) Then, an MTT cytotoxicity assay was performed. MTT in RPMI medium in final concentration 0.3 mg/ml was used. The formazan crystals were diluted in DMSO after 30-40 min of incubation. Then, the absorbance was measured at 595 nm with Infinite M200 (TECAN, Austria).
b) Thereafter, a Crystal violet proliferation assay was performed. The medium was replaced with Crystal violet solution (0.5 %; v/v) and incubated on Rocker-Shaker (Biosan) for 1 h. After that, the aqueous solution was removed and the cells were washed five times with 1x PBS. The dye was dissolved in 200 µl of methanol and measured at 595 nm with Infinite M200 (TECAN, Austria).

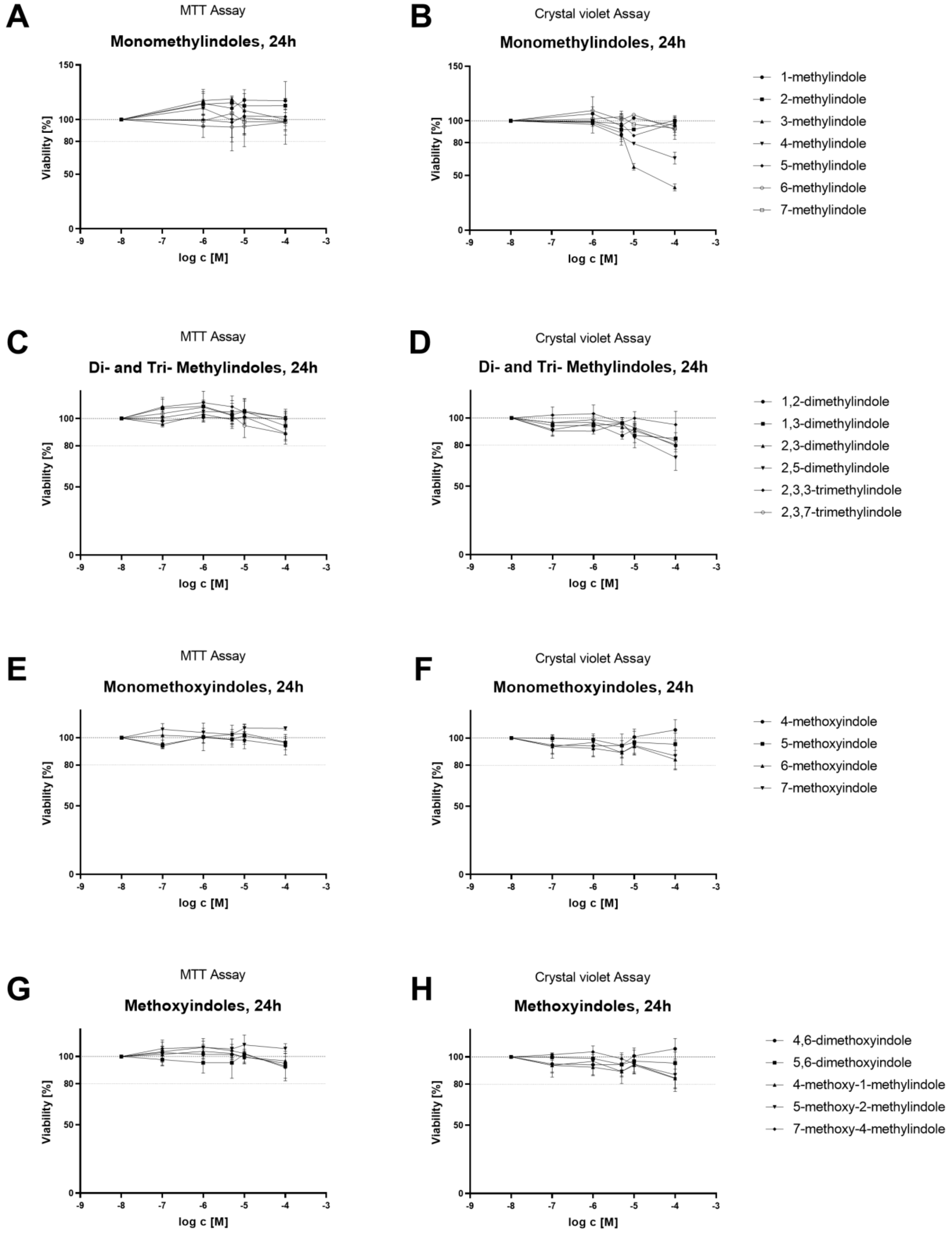

Supplement S2: Cytotoxicity of tested indoles. 22Rv1 cells were incubated for 24 with indoles in the concentration range from 0.1 μM to 100 μM or controls (UT; doxorubicin 20 μM). The cytotoxic effects were then evaluated using MTT or crystal violet assays. All 22 tested indoles were divided into groups titled as Monomethylindoles (A, B), Di- and Tri-Methylindoles (C, D), Monomethoxyindoles (E, F), and Methoxyindoles (G, H). The MTT assay results are given in the left column and the crystal violet assay results are given in the right column. Results are expressed in % of untreated (UT; DMSO-treated) cells.

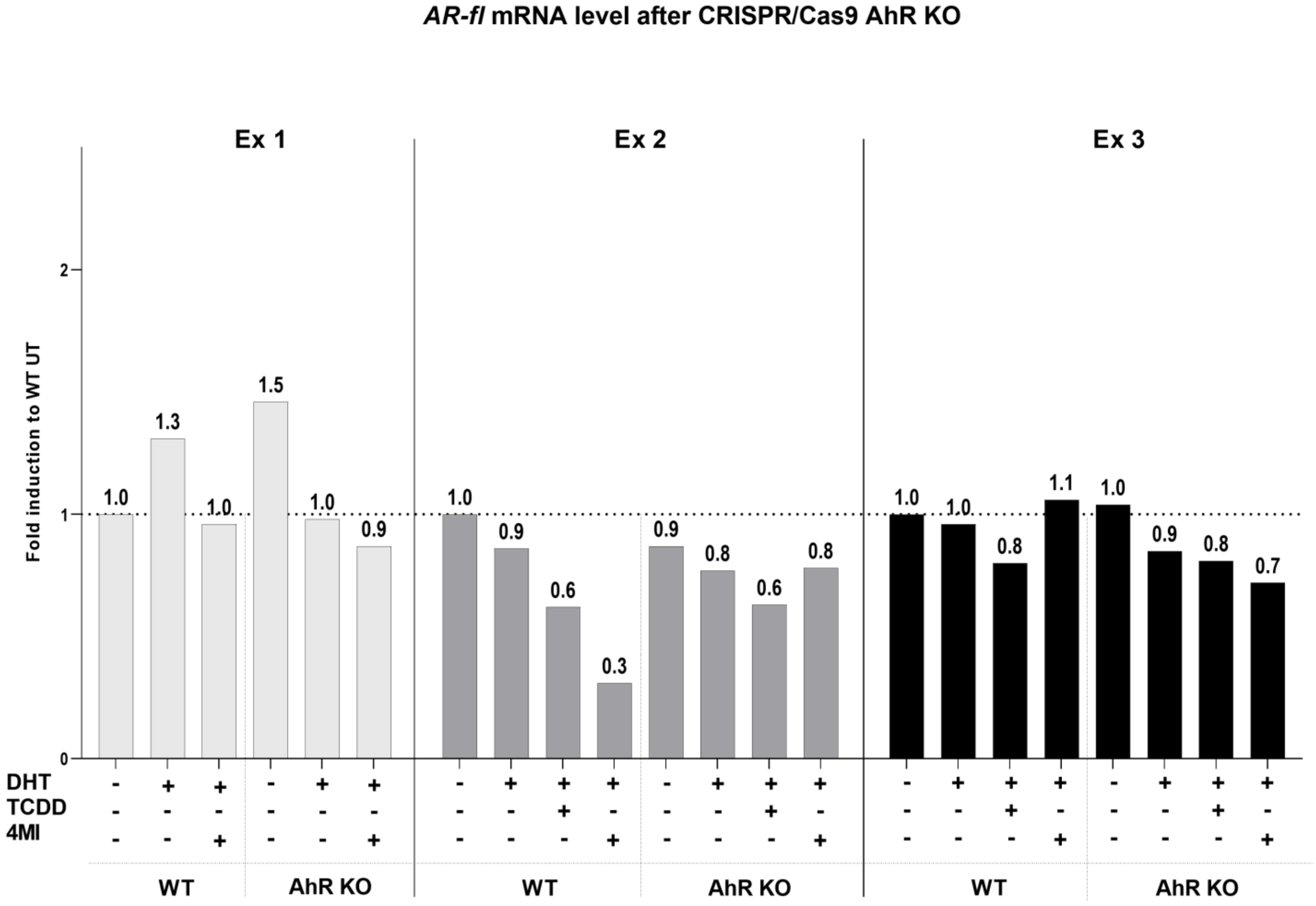

Supplement S3. Expression of *AR-fl* mRNA CRISPR/Cas9 AhR knockout. 22Rv1 cells were transiently transfected with the CRISPR/Cas 9 AhR knockout plasmid or the Control CRISPR/Cas9 plasmid. After 48 h, transfected cells were treated with controls (UT; TCDD 10 nM; DHT 10 nM) and 4 MI 100 µM. The induction of *AR-fl* was determined by RT-qPCR. The data obtained were normalized per housekeeping gene *GAPDH*. The results are expressed in fold induction to wild-type UT. In total, 3 independent experiments were analysed.

Supplement S4: Key structures of tested indoles

Compound	Molecular formula	Structure
**1-methylindole**	C_9H_9N	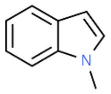
**2-methylindole**	C_9H_9N	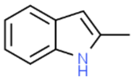
**3-methylindole**	C_9H_9N	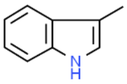
**4-methylindole**	C_9H_9N	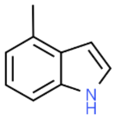
**5-methylindole**	C_9H_9N	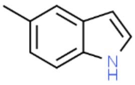
**6-methylindole**	C_9H_9N	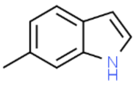
**7-methylindole**	C_9H_9N	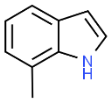
**1,2-methylindole**	$C_{10}H_{11}N$	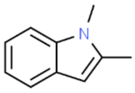
**1,3-methylindole**	$C_{10}H_{11}N$	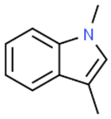
**2,3-methylindole**	$C_{10}H_{11}N$	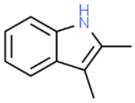
**2,5-methylindole**	$C_{10}H_{11}N$	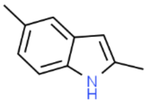
**2,3,3-trimethylindolenine**	$C_{11}H_{13}N$	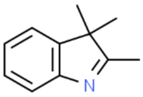

**2,3,7-trimethylindole**	$C_{11}H_{13}N$	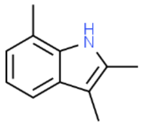
**4-methoxyindole**	C_9H_9NO	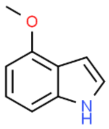
**5-methoxyindole**	C_9H_9NO	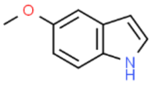
**6-methoxyindole**	C_9H_9NO	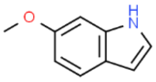
**7-methoxyindole**	C_9H_9NO	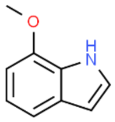
**4,6-dimethoxyindole**	$C_{10}H_{11}NO_2$	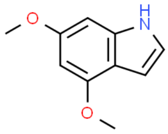
**5,6-dimethoxyindole**	$C_{10}H_{11}NO_2$	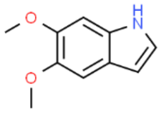
**4-methoxy-1-methylindole**	$C_{10}H_{11}NO$	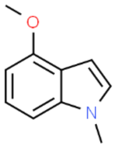
**5-methoxy-2-methylindole**	$C_{10}H_{11}NO$	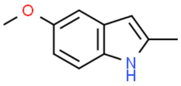
**7-methoxy-4-methylindole**	$C_{10}H_{11}NO$	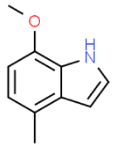

Supplement S5: List of used chemicals.

Product	Manufacturer	Catalog #	Lot / Batch #
**DMSO**	Sigma	D4540	BCCB6907
**DMSO pure for MTT**	Lach-Ner	20022-CT0	PP/2020/01579
**Dulbecco's PBS**	Serana	BDL-001	01070619BDL, 01071721BDL, 01041720BDL
**Ethyl Alcohol, 99,8%**	Lach-Ner	20025-A99	PP/2017/09915
**Fetal Bovine Serum**	Sigma Aldrich	F7524	1640522, 1638262
**Hygromycin B**	Santa cruz Biotechnology	sc-29067	H2319
**L-Glutamine**	Serana	RGL-001	01061019RGL, 13100619RGL
**MEM Non-essential amino acid solution**	Sigma Aldrich	M7145	RNBJ0369, RBNK8956
**Methyl Alcohol**	Penta Chemicals	21210-20005	2505130520
**MycoAlert Mycoplasma Detection Kit**	Lonza	LT07-118	921989
**RPMI-1640**	Sigma Aldrich	R0883	RNBH9726, RNBK9935, RNBL0672, RNBK0723
**Trypan Blue cell culture tested**	Sigma	T9650	SLBV4976
**Trypsin 0.25% - EDTA in HBSS**	Biosera	MS00CY100B	LM-T1720/500
**Water, Nuclease-free**	Sigma Aldrich	W4502	1003379140
Reporter Gene Assay	Manufacturer	Catalog #	Lot / Batch #
**Adenosine 5'-triphoshpate disodium salt hydrate**	Sigma Aldrich	A2383-5G	SLBD2725V
**Coenzyme A sodium salt hydrate**	Sigma Aldrich	C4780-100MG	SLCB8868
**D-Luciferin**	Sigma Aldrich	L9504-50MG	SLBS2450V
**DTT**	Sigma Aldrich	43819	BCCB7858
**Ethylenediaminetetraacetic Acid**	Sigma Aldrich	E9884	BCBN2979V
**MgSO4 x 7 H2O**	LACHEMA		403
**Reporter Lysis 5x Buffer**	Promega	E3971	329638, 413170, 398845
**Tris-Acetate-EDTA Buffer**	Sigma Aldrich	T9650	SLBV4976
Cytotoxicity Assays	Manufacturer	Catalog #	Lot / Batch #
**Crystal Violet 1%**	Sigma Aldrich	V5265-500ML	SCLF5128
**Doxorubicin**	PromoCell GmbH	PK-CA577-K329	452PO98-1
**Thiazolyl Blue Tetrazolium Bromide (MTT)**	Sigma Aldrich	MT2128	MKCD8033
RNA Isolation and Reverse Transcription	Manufacturer	Catalog #	Lot / Batch #
**10x M-MuLV Reaction Buffer**	BioLabs ® Inc.	B0253S	10058397, 10104588
**dATP (2'-deoxyadenosine 5'-triphosphate)**	TaKaRa	4026	B7701A
**dCTP (2'-deoxycytidine 5'-triphosphate)**	TaKaRa	4028	B7901A
**dGTP (2'-deoxyguanosine 5'-triphosphate)**	TaKaRa	4027	B8001A
**dTTP (thymidine 5'-triphosphate)**	TaKaRa	4029	B7501A
**Chloroform**	Sigma Aldrich	C2432	SHBD5858V

**Isopropyl Alcohol**	Lach-Ner	20037-AT0	PP/2017/03711
**M-MuLV (reverse transcriptase)**	BioLabs ® Inc.	M0253L	10099097, 10111985
**Random Primers 6**	BioLabs ® Inc.	S1230S	10058397, 1014737, 10102123
**RNAse inhibitor**	BioLabs ® Inc.	M0307L	10115397
**Tri Reagent ® - RNA/DNA/Protein isolation reagent**	Molecular Research Center, inc	TR118	7111
**RT-qPCR**	**Manufacturer**	**Catalog #**	**Lot / Batch #**
**AhR Forward Primer (FH1_AhR)**	Sigma Aldrich	8815864959-210/0	ST04825127-001
**AhR Probe (PH1_AhR)**	Sigma Aldrich	8815864959-210/2	ST04825129-002
**AhR Reverse Primer (RH1_AhR)**	Sigma Aldrich	8815864959-210/1	ST04825128-001
**AhRR Forward Primer (FH1_AhRR)**	Sigma Aldrich	8815864959-260/0	ST04825142-001
**AhRR Probe (PH1_AhRR)**	Sigma Aldrich	8815864959-260/2	ST04825144-002
**AhRR Reverse Primer (RH1_AhRR)**	Sigma Aldrich	8815864959-260/1	ST04825143-001
**AR Forward Primer (FH1_AR)**	Sigma Aldrich	8815864959-150/0	ST04825109-001
**AR Probe (PH1_AR)**	Sigma Aldrich	8815864959-150/2	ST04825111-002
**AR Reverse Primer (RH1_AR)**	Sigma Aldrich	8815864959-150/1	ST04825110-001
**AR v7 Forward Primer (FH1_ARv7)**	Sigma Aldrich	8816914473-000010	SY210541259-025
**ARv7 Reverse Primer (RH1_ARv7)**	Sigma Aldrich	8816914473-000020	SY210541259-026
**CYP1A1 Forward Primer (FH1_CYP1A1)**	Sigma Aldrich	8815746405-10/0	ST04795193-004
**CYP1A1 Probe (PH1_CYP1A1)**	Sigma Aldrich	8815746405-10/2	ST04795194-004
**CYP1A1 Reverse Primer (RH1_CYP1A1)**	Sigma Aldrich	8815746405-10/1	ST04795192-004
**FKBP5 Forward Primer (FH1_FKBP5)**	Sigma Aldrich	8815864959-280/0	ST04825148-001
**FKBP5 Probe (PH1_FKBP5)**	Sigma Aldrich	8815864959-280/2	ST04825150-002
**FKBP5 Reverse Primer (RH1_FKBP5)**	Sigma Aldrich	8815864959-280/1	ST04825149-001
**GAPDH Forward Primer (FH1_GAPDH)**	Sigma Aldrich	8817264953-40/0	ST04780378-001
**GAPDH Probe (PH1_GAPDH)**	Sigma Aldrich	8817264953-40/2	ST04780380-002
**GAPDH Reverse Primer (RH1_GAPDH)**	Sigma Aldrich	8817264953-40/1	ST04780379-001
**KiCqStart Probe qPCR ReadyMix**	Sigma Aldrich	KCQS04	LN Q66191473, LN Q66184201
**KLK3 Forward Primer (FH1_KLK3)**	Sigma Aldrich	8815864959-240/0	ST04825136-001
**KLK3 Probe (PH1_KLK3)**	Sigma Aldrich	8815864959-240/2	ST04825138-002
**KLK3 Reverse Primer (RH1_KLK3)**	Sigma Aldrich	8815864959-240/1	ST04825137-001
**LightCycler ® 480 SYBR® Green I Master**	Roche	4707516001	
**UBE2C Forward Primer (FH1_UBE2C)**	Sigma Aldrich	8815864959-270/0	ST04825145-001
**UBE2C Probe (PH1_UBE2C)**	Sigma Aldrich	8815864959-270/2	ST04825147-002
**UBE2C Reverse Primer (RH1_UBE2C)**	Sigma Aldrich	8815864959-270/1	ST04825146-001
**Protein Isolation**	**Manufacturer**	**Catalog #**	**Lot / Batch #**
**HEPES**	Sigma Aldrich	H3375	BLBX2657

**Bradford Reagent**	Sigma Aldrich	B6916	SLCK1933
**Halt Protease Inhibitor Single-Use Cocktail**	ThermoFisher Scientific	78430	VG297201
**PhosSTOP EASYpack**	Roche	4906837001	62344300
**Sodium chloride**	LachNer	30093-AP0	PP/2014/02150
**Triton X-100**	Sigma Aldrich	T9284	1003426861
**Western Blotting**	**Manufacturer**	**Catalog #**	**Lot / Batch #**
**Re-Blot Plus Strong Solution (10x)**	Sigma Aldrich	2504	3286474, 3614274
**10x Tris/Glycine Buffer**	BioRad	1610771	64337786
**10x Tris/Glycine/SDS Buffer**	BioRad	1610732	64467353
**20x TBS Buffer**	ThermoFisher Scientific	28358	WD322613
**Acetic acid, 99.8%**	Penta Chemicals	607-002-00-6	1812061213
**Acrylamide/Bis-acrylamide**	Sigma Aldrich	A2792	SLCJ1949
**Ah Receptor antibody (A-3)**	Santa cruz Biotechnology	sc-133088	K0920
**Amonium Persulfate, for electrophoresis**	Sigma Aldrich	A3678	MKBC7986
**Anti-mouse HRP-linked antibody**	Cell Signaling	7076S	36
**AR antibody (441)**	Santa cruz Biotechnology	sc-7305	A2020
**Bovine Serum Albumin**	Sigma Aldrich	A2153	SCJ7629
**Bromphenol Blue**	Sigma Aldrich	B0126	79696HJV
**Glycerol**	Lach-Ner	40058-A50	PP/2017/02241
**Immobilon® -P Transfer Membrane**	Sigma Aldrich	IPVH00010	180320
**Non-fat dried milk Laktino**	ARTIFEX Instant s.r.o.		CZ 17847 ES
**Ponceau S. solution**	Santa cruz Biotechnology	sc-301558	A0421
**Running buffer**	BioRad	1610798	L004167A
**Sodium Dodecyl Sulfate**	Sigma Aldrich	L3771	SLBH870V
**Stacking buffer**	BioRad	1610799	L004168A
**TEMED**	Sigma Aldrich	110732	K51567332.928
**Tween 20**	Sigma Aldrich	P1379	SZBC1240V
**WesternSure ® PREMIUM Chemiluminescent Substrate**	Li-Cor	926-95000	VH311910, VI311909, UH293688, UI293689
**WesternSure® Pre-Stained Chemiluminiscent Protein Ladder**	Li-Cor	926-98000	D10827-01, D00227-01
**Chromatin Immunoprecipitation**	**Manufacturer**	**Catalog #**	**Lot / Batch #**
**100bp DNA Ladder**	Promega	G210A	456865
**Agarose**	Serva	11380.02	170138
**Agarose, LMP**	Promega	V3841	336732
**Androgen Receptor D6F1I (rabbit mAb)**	Cell Signaling	5153S	9
**Anti-rabbit IgG**	Cell Signaling	7074P2	29

**Blue/Orange 6x Loading Dye**	Promega	G190A	455992
**GelRed® Nucleic Acid Stain**	Biotium	41003	21G1020
**ChIP-Grade Protein G Magnetic Beads**	Cell Signaling	9006	25
**Micrococcal Nuclease**	Cell Signaling	10011S	21
**Proteinase K**	ThermoFisher Scientific	4333793	10860221
**SimpleChIP® Plus Enzymatic Chromatin IP Kit**	Cell Signaling	9005	
**SimpleChIP® KLK3 Promoter Primers**	Cell Signaling	32784S	1
**Ultra Low Range DNA Ladder**	Invitrogen	10597012	538912
**Transfection and CRISPR/Cas9 reagents**	**Manufacturer**	**Catalog #**	**Lot / Batch #**
**FuGENE ® HD - transfection reagent**	Promega	E231A	216863
**Ah Receptor CRISPR/Cas9 KO Plasmid (h)**	Santa Cruz Biotechnology	sc-400297	F2619
**Control CRISPR/Cas9 Plasmid**	Santa Cruz Biotechnology	sc-418922	J2120
**Plasmid Transfection Medium**	Santa Cruz Biotechnology	sc-108062	J1920
**UltraCruz® Transfection Reagent**	Santa Cruz Biotechnology	sc-395739	H1220

Compound	Manufacturer	Catalog #	Lot / Batch #
**1-methylindole**	Sigma Aldrich	193984	BGBC4251V
**2-methylindole**	Sigma Aldrich	M51407	BCBF7494V
**3-methylindole**	Sigma Aldrich	M51458	STBK2264
**4-methylindole**	Energy Chemical	E020483	AH160074
**5-methylindole**	Sigma Aldrich	222410	STBF3756V
**6-methylindole**	Sigma Aldrich	246328	MKBPJ2514V
**7-methylindole**	Sigma Aldrich	M51490	MKBP5287V
**1,2-di-methylindole**	Sigma Aldrich	D165603	S43286V
**1,3-di-methylindole**	Shanghai SINKH Pharmaceuticals Tech. Co., Ltd.	875-30-9	
**2,3-di-methylindole**	Sigma Aldrich	120812	STBF0465V
**2,5-di-methylindole**	Sigma Aldrich	D166006	1408950V
**2,3,3-tri-methylindolenine**	Sigma Aldrich	T76805	STBF9768V
**2,3,7-tri-methylindole**	Sigma Aldrich	CDS014194	B02665235
**4-methoxyindole**	Sigma Aldrich	246298	STBF5169V
**5-methoxyindole**	Sigma Aldrich	M14900	10206LHV
**6-methoxyindole**	Sigma Aldrich	139858	MKBD1228V
**7-methoxyindole**	Sigma Aldrich	SC254908	1430903V
**4,6-di-methoxyindole**	Sigma Aldrich	75066-1G-F	BCBF8458V
**5,6-di-methoxyindole**	Sigma Aldrich	246255	MKD30455V
**4-methoxy-1-methylindole**	Sigma Aldrich	259055	08827AD

**5-methoxy-2-methylindole**	Sigma Aldrich	M15451	STBD7007V
**7-methoxy-4-methylindole**	1ClickChemistry Inc	1C72757	55484A
**BaP (Beno[a]pyrene)**	Sigma Aldrich	B1760	
**DHT (5α-Androstan-17β-ol-3-one)**	Sigma Aldrich	A8380	
**ENZ (MDV3100)**	Santa cruz Biotechnology	sc-364354	I1021
**FICZ (6-Formylindolo[3,2-b]carbazole)**	Sigma Aldrich	SML1489	26019
**TCDD**	Ultra Scientific	RPE-029	

References:

1. Novotna, A.; Pavek, P.; Dvorak, Z. Novel stably transfected gene reporter human hepatoma cell line for assessment of aryl hydrocarbon receptor transcriptional activity: Construction and characterization. *Environ Sci. Technol.* **2011**, *45*, 10133–10139. https://doi.org/10.1021/es2029334.
